# Mathematical modeling of weight loss and crust temperature of toast bread containing guar gum during baking process

**DOI:** 10.1002/fsn3.1993

**Published:** 2020-12-04

**Authors:** Farnaz Mohammadi Golchin, Sara Movahhed, Mohammadreza Eshaghi, Hossein Ahmadi Chenarbon

**Affiliations:** ^1^ Department of Food Science and Technology, College of Agriculture, Varamin ‐ Pishva Branch Islamic Azad University Varamin Iran; ^2^ Department of Agronomy, College of Agriculture, Varamin ‐ Pishva Branch Islamic Azad University Varamin Iran

**Keywords:** bread baking, bread crust temperature, bread weight loss, mathematical model, moisture content

## Abstract

Baking is a combined process in which several interconnected chemical, biochemical, and physical phenomena such as starch gelatinization and formation of porous structures occur. These changes affect the ultimate quality of the product such as taste. In this study, effects of guar gum (at 1% level) and baking temperature (at 190, 200, 210, 220, and 230°C) on crust temperature and weight loss of toast bread samples during 40 min exposure were investigated. According to the results, raising the oven's temperature from 190°C to 230°C will rise the crust's temperature of toast bread samples both in the control group (from 128.5°C to 190.2°C) and test group (from 120.18°C to 164.8°C), as well as the percentage of weight loss in toast bread samples both in the control group (from 35.11% to 47.23%) and test group (from 20.37% to 29.57%). We also worked with polynomial functions, exponential functions, fractional functions, Gaussian functions, and MATLAB to model the percentage of weight loss and an increase in the crust temperature of toast bread samples during the baking process. And ultimately, the correlation coefficients of the models were calculated and compared to analyze the results of predictor functions.

## Graphical abstract

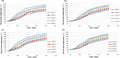

## 1 INTRODUCTION

In Iran, on average, more than half of the energy and protein consumed by households originates from bread. As with studies conducted, every low‐income citizen receives 60% of energy and 67% of protein from bread consumption, with corresponding figures of 66% and 72% for rural areas (Movahhed, 2017; Movahhed et al., 2014). Generally, bread is classified into three groups by volume including bulk, semibulk, and flatbreads. Toast bread belongs to the group of bulk breads and is usually consumed for breakfast in most regions of the world. The waste and scraps of this bread are less than many other types if they are consumed freshly and avoided from staling. Importantly, the quality of bulk breads depends on the baking capacity of the flour, duration of fermentation, protein content, and type of additives. Besides, the baking capacity of the flour largely depends on the characteristics of the flour, the type of industrial action, the method of preparation, and the preparation of the dough (Movahhed et al., 2014). During the baking of bread, heat transfer in dough is a combination of conduction from band or tins, convection from the surrounding hot air, radiation from the oven walls to the product surface, conduction in the continuous liquid/solid phase, and evaporation of water and condensing steam in the gas phase of the dough (Wagner et al., 2007). Baking is the foremost step in making bread, during which a set of physical, chemical, and biochemical changes occur in the product. And heat and gas expansion, browning, deformation of protein status, colorization, starch gelatinization, the formation of porous structure, and crust formation are the main outcomes. In accordance with the former studies, the interaction between starch and protein in bread has a significant impact on staling the bread. During the process of baking bread, a large amount of starch gelatinizes and even the oven temperature can make the starch to dissolve. Taking into account that starch molecules interact with protein molecules, it is probable that more interaction has occurred in samples with more dissolved starch. According to the results of the research, bread with a longer baking time at a lower temperature includes less dissolved starch than bread with shorter baking time at higher temperatures. Thus, as already mentioned determining the most suitable oven temperature is of considerable importance. Improper bread baking can result in doughy texture, unpleasant color, taste, or odor, burn, early staling of bread, all of which can be caused by thermal processes such as nonenzymatic browning, starch gelatinization, and denaturation (Bellido et al., 2009; Cato et al., 2004; Mondal & Datta, 2010). These thermal processes occur through heat and mass transfer mechanisms within the bread and baking medium. A precise understanding of the process of heat transfer during baking the various types of bread can lead to solutions such as changing the thermodynamic and radiant properties of the internal walls, changing the geometric shape of the oven, determining the suitable spot for placing bread inside the oven, determining the proper speed of movement of the gases inside the oven in order to improve the quality of bread and decrease the energy consumption (Movahhed & Ahmadi Chenarbon, 2018). Therefore, to analyze and optimize the baking process, heat and mass transfer parameters (e.g. effective heat diffusion, heat transfer coefficient, effective moisture diffusion, moisture or mass transfer coefficient, and conduction heat transfer coefficient of the product) need to be investigated (Bellido et al., 2009; Collar et al., 2009). The heat transfer coefficient, for example, is a principal parameter contributing to maintaining the quality of the product after baking. The heat transfer coefficient during the raising phase plays the central role in improving the sensory properties of the product, stimulating the browning (Millard reaction) and caramelizing reaction, which both cause the full diffusion of flavor, color, and texture of the food material. The quality of the crust formed during the raising phase is an important factor with the optimal heat transfer rate and texture of the product. Measurement of the heat transfer coefficient, therefore, is essential to understand the complexities of the baking process. However, the uneven distribution of temperature between the surface of the product and its interior parts (crumb) makes heat transfer coefficients difficult to estimate (Farkas & Hubbard, 2000; Toufeili et al., 2009). As a result, studying the baking process to produce a high quality product and also the conservation of energy seem to be essential. Various and simplified mathematical models have been developed so far to describe the baking process that can help us to simulate heat and mass transfer phenomena during baking (Bikard et al., 2008; Purlis, 2010, 2012; Sakin et al., 2009; Zhang & Datta, 2006). But as stated, baking is a complex process where the bread quality is affected by the baking medium conditions, internal processes, and the process of heat and mass transfer within the bread. Therefore, to achieve proper baking conditions, the baking medium and the internal processes of bread should also be considered. Therefore, the kinetic models of the baking process need to be solved, besides the external heat transfer models and the internal heat and mass transfer models. Generally, the internal kinetic processes of bread are highly complex and the mathematical models proposed for them are highly influenced by the type of flour and the dough processing, many of which are still unknown. Thus, a model that intends to calculate the qualitative parameters and apply them in modeling seems to come with some inefficiency (Sakin et al., 2009; Toufeili et al., 2009). Certainly, a full understanding of the kinetic parameters derived from the modeling of the baking process enables us to predict end‐product qualitative changes and improve process conditions. In this regard, several models have been reported at various levels of complexity to describe the phenomenon of moisture loss during the baking process. For example, a first‐order kinetic model has been proposed where the rate of moisture loss is assumed to be proportional to the moisture content of the material. Also, in a more complex model, the product is deemed to consist of two crustal and boundary regions separated by a movable (changeable) boundary. Conversely, other researchers have worked with Fick's Second Law of Diffusion to model the mass transfer phenomenon. This law gives a simple illustration of moisture loss during the baking process, which is consistent with experimental data (Sablani et al., 1998; Troncoso & Pedreschi, 2009). Because of the complexity, multiple assumptions have been proposed to simplify these models. A general assumption in this respect is that food processing occurs under isothermal conditions, which fixes the problem of parallel transfer of moisture and heat. Also, according to the rule of sum, we can describe how a phenomenon occurs by knowing the geometry of an object. Accordingly, the geometric shape of the bread in the form of rectangular cubes can be assumed to be a restricted plate that results from the collision of two unlimited plates (Movahhed & Ahmadi Chenarbon, 2018; Pastukhov & Danin, 2011; Yildiz et al., 2007).

As with the foregoing topics, understanding and interpreting heat and mass transfer phenomena as foremost parameters are critical to maintaining the quality of baking products and, also, the optimal design of baking equipment because the application of improper heats in unusual ways destroys the sensory properties of the product, the flavor, the color and texture of the food, as well as wasting a great deal of energy. Among the related researches, no one has ever studied the effect of baking time and baking temperature on crust temperature and weight loss of toast bread containing guar gum during baking process. Therefore, doing research on this topic seems to be necessary. However, this research is following two goals: (a) studying the effect of the baking time, baking temperature, and guar gum on crust temperature and weight loss of toast bread during baking process, and (b) developing a mathematical model for predicting the crust temperature and weight loss of toast bread at different oven temperatures and baking times.

## 2 MATERIALS AND METHODS

### 2.1 Materials

Materials utilized in the study including wheat flour, suitable for the making of Toast bread with the extraction grade of 78%, (Alborz Flour Co), Guar Hydrocolloid (Tellon Co), Yeast (*Saccharomyces cerevisiae*) (Iran Malas Co), Salt (Sadaf Co), Sugar (Etminan Co), and Oil (Bahar Co) were first purchased.

### 2.2 Methods

#### 2.2.1 Preparation of bread samples

To make the toast bread, guar gum was first added to wheat flour at a rate of 1% and evenly mixed in the dough kneader for 10 min, and then the other ingredients were added to each mixture. Afterward, the water was added to the mixture and mixed by a stirrer at 750 rpm for 3 to 7 min. After the dough was formed, the samples were kept intact for 10 min and kneaded to form dough balls of ca. 450 g. Inner fermentation was then performed after 10 min of rest. Finally, the dough balls were entered into the fermentation chamber for 40 min for final fermentation at the temperature of 30°C and the relative humidity of 80%. The prepared dough is put in oven (BOSCH, HBA 73 DD550) at different temperatures (190, 200, 210, 220 and 230°C) on hot aluminum slab with 1 cm thickness in temperature equilibrium with the oven's temperature and they were taken out from the oven at certain time intervals (5, 10, 15, 20, 25, 30, 35 and 40 min). Moisture content of Toast's dough was evaluated during baking according to the AACC method 44‐15A with an air oven (Memmert, UNB400). Also, the parameters used in equations are presented in Table 1.

**TABLE 1:** Input parameters used in modeling of toast bread baking

Parameter	Unit	Value	Reference
Water vapor pressure at saturation, *P_sat_*	Pa	0.98 × 10^5^	Khater & Bahnasawy, 2014
Stefan–Boltzmann constant, *σ*	W m^2^K^−4^	5.67 × 10^–8^	Khater & Bahnasawy, 2014
Relative humidity, *RH*	%	10	This study
Initial temperature	K	293	This study
Initial water constant, *W*, control	Dry basis	0.58	This study
Initial water constant, *W*, containing guar gum	Dry basis	0.62	This study
Bread radius, *r*	m	0.061	This study
Initial dough density, control, *ρ*	Kg m^−3^	402	This study
Initial dough density, containing guar gum, *ρ*	Kg m^−3^	421	This study
Emissivity, ε	‐	0.9	Hamdami et al., 2004
Delta‐type function, δ	‐	1	Khater & Bahnasawy, 2014
Heat transfer coefficient, *h*	W/m^2^ K^−1^	10	Purlis, 2012
Density of solid matrix, control, *ρ_s_*	Kg m^−3^	315	This study
Density of solid matrix, containing guar gum, *ρ_s_*	Kg m^−3^	341	This study
Latent heat of evaporation, λ_v_	J/kg	2.33 × 10^6^	Purlis, 2012
Water diffusion coefficient, *D*	m^2^s^−1^	12 × 10^–5^	This study
Kinetic viscosity, *v*	m^2^s^−1^	28.86 × 10^–6^	Magee & Branshurg, 1995
Thermal diffusivity, α	m^2^s^−1^	1.17 × 10^–7^	Magee & Branshurg, 1995
Specific heat of bread, control, *C_P_*	J/kg K^−1^	2,489	This study
Specific heat of bread, containing guar gum, *C_P_*	J/kg K^−1^	1,640	This study
Bread thermal conductivity, control, *k*	W/m K^−1^	0.18	This study
Bread thermal conductivity, containing guar gum, *k*	W/m K^−1^	0.28	This study

#### 2.2.2 Model development

In modeling, the bread can be divided into three areas, including (a) Crumb, which is the central region, where the temperature does not exceed 100°C, (b) Crust, which is the outermost dry layer with temperatures exceeding 100°C, where the samples lose their water); and (c) Evaporation Front, which is between the central area and the crust, where the temperature is 100°C and evaporation occurs. The following are some assumptions for a better analysis of the process.

- The volume changes are negligible.
- Bread is a homogenous and interconnected medium.
- Heat is transmitted by conduction from the crust to the center of the bread as with the Fourier law.
- Water infiltration occurs only in the humid inner area (Crumb) while the vapor diffusion occurs only in the crustal area.
- Energy is transferred from the oven to the surface of the bread by convective flux and irradiation.
- Water diffusion from the center to the evaporation front occurs due to water flux caused by the water content gradient, which can be described by Fick's diffusion law.
- The diffusion of water vapor from the evaporation front to the crust occurs because of the water flux caused by the water vapor concentration gradient, which can be described by Fick's diffusion law.
- Water vapor is diffused to the oven medium by the convective flux (Purlis, 2012; Purlis & Salvadori, 2009).

#### 2.2.3 Governing equations

In the first step, the bread was considered to be an infinite cylinder with radius r and heat transfer was investigated only in one direction due to a single dimension via the axial symmetry assumption. Temperature and moisture content was assumed to be the same for initial conditions.

##### Heat balance equation

(1) $$\rho C_{P}\frac{\partial T}{\partial t}=\frac{1}{r}\frac{\partial }{\partial r}\left(rk\frac{\partial T}{\partial r}\right)$$

The heat diffuses to the crust of the bread by convective flux and irradiation, while it is diffused within the bread by conduction.

##### Boundary condition

(2) $$‐k\frac{\partial T}{\partial r}=h\left(T_{s}‐T_{\infty }\right)+\varepsilon \sigma \left(T_{s}^{4}‐T_{\infty }^{4}\right)$$

##### Mass balance equation

(3) $$\frac{\partial W}{\partial t}=\frac{1}{r}\frac{\partial }{\partial r}\left(rD\frac{\partial W}{\partial r}\right)$$

Also, the diffusion of water to the crust by a convective flux leading to a balance in water spread.

##### Boundary conditions

(4) $$‐D\rho_{s}\frac{\partial W}{\partial t}=k_{g}\left(P_{s}\left(T_{s})‐P_{\infty }(T_{\infty }\right)\right)$$

(5) $$P_{s}\frac{\partial T}{\partial t}=a_{w}P_{\mathrm{sat}}\left(T_{s}\right)$$

(6) $$P_{\infty }=\frac{\mathrm{RH}}{100}P_{\mathrm{sat}}\left(T_{\infty }\right)$$

(7) At the center,i.e.r=0

(8) $$\frac{\partial T}{\partial t}=0$$

(9) $$\frac{\partial W}{\partial t}=0$$

##### Thermo‐physical properties

###### Specific heat

(10) $$C_{P}\left(T,W\right)=C_{p}^{*}\left(T,W\right)+\lambda_{v}W\delta$$

(11) $$C_{p}^{*}\left(T,W\right)=C_{p,s}\left(T\right)+WC_{p,w}\left(T\right)$$

(12) $$C_{p,s}=5\left(T\right)+24$$

(13) $$C_{p,w}=\left(5.207‐73.17\times 10^{‐4}T+1.35\times 10^{‐5}10^{2}\right)\times 1000$$

The latent heat of vaporization was calculated from Equation (14) (Bikard et al., 2008; Purlis & Salvadori, 2009).

(14) $$\lambda_{v}=\left(2502535.259‐212.56384\left(T‐273\right)\right)$$

###### Water activity

(15) $$a_{w}\left(T,W\right)={\left({\left(\frac{100W}{\exp\left(‐0.0056T+55\right)}\right)}^{\frac{‐1}{0.38}}+1\right)}^{‐1}$$

In the simulation, the heat transfer coefficient (*h*) was considered as the input of the model and the mass transfer coefficient was determined by the Chilton–Colburn analogy and its correction factor.

(16) $$\frac{h}{k_{g}^{*}}=\frac{M_{\mathrm{air}}}{M_{w}}P_{\mathrm{atm}}C_{p,air}{\left(\frac{S_{c}}{\mathrm{Pr}}\right)}^{\frac{2}{3}}$$

(17) $$k_{g}=7.83\times 10^{‐3}k_{g}^{*}$$

(18) $$S_{c}=\frac{\upsilon }{D}$$

(19) $$Pr=\frac{\upsilon }{\alpha }$$

###### Determination of specific heat capacity (SHC)

In this study, the mixing method was adopted to measure the specific heat capacity of bread samples (Shrivastava & Datta, 1999; Subramanian & Viswanathan, 2003). And the following assumptions were also made for testing purpose: (a) Heat dissipation from the hot water container to the calorimeter during the transfer of capsule containing the test sample is negligible. (b) At the end of the heating of the capsule and sample, the temperature of the test sample and capsule is the same. (c) The evaporation in the calorimeter during the time to reach thermal equilibrium is negligible. To conduct tests, some instruments were used including aluminum cylinder (15.2 mm in diameter, 52.6 mm in height and 2.1 mm in thickness) for holding (storing) samples, T‐type thermocouple equipped with a thermal display, a thermal isolation flask with a capacity of 250 cm^3^ and an oven. To determine the heat capacity of the calorimeter, a certain volume of high‐temperature (up to 70°C) distilled water was poured into the calorimeter containing a certain volume of low‐temperature distilled water. Then, the heat capacity of the calorimeter was calculated by Equation (20).

(20) $$H_{f}=\frac{M_{\mathrm{cw}}C_{w}\left(T_{e}‐T_{\mathrm{cw}}\right)‐M_{\mathrm{hw}}C_{w}\left(T_{\mathrm{hw}}‐T_{e}\right)}{\left(T_{\mathrm{hw}}‐T_{e}\right)}$$

To determine the heat capacity of the aluminum capsule, the high‐temperature capsule was placed in a calorimeter containing a given volume of distilled water at low temperature (room temperature). The system was assumed to be adiabatic. Then the heat capacity of the capsule was determined using Equation (21).

(21) $$H_{c}=\frac{\left(H_{f}+M_{\mathrm{cw}}C_{w}\right)\left(T_{e}‐T_{\mathrm{cw}}\right)}{\left(T_{c}‐T_{e}\right)}$$

Then, to measure the specific heat capacity of the bread samples, the aluminum capsule was filled with the samples and placed in the oven at the test temperature for one hour. The test capsule was then placed in a calorimeter with a given volume of distilled water at low temperature to reach the thermal equilibrium and then the equilibrium temperature was recorded. The SHC of the bread samples was then measured using Equation (22).

(22) $$C_{p}=\frac{\left(H_{f}+M_{\mathrm{cw}}C_{w}\right)\left(T_{e}‐T_{\mathrm{cw}}\right)‐H_{c}\left(T_{m}‐T_{e}\right)}{M_{m}\left(T_{m}‐T_{e}\right)}$$

###### Determination of thermal conductivity

To measure the thermal conductivity of the bread samples, the linear heat source (Hot Wire) method was used (Casada & Walton, 1989). Equipment used consisted of a brass cylinder (240 mm in the high and internal diameter of 58.6 mm) equipped with a removable rubber cap at the top and a fixed rubber door at the bottom. The heat source was a Constantine thermal wire with a diameter of 0.32 mm and a length of 235 mm (11.49 Ω) that was connected to a direct current source. The current rate was regulated by a rheostat. Also, a T‐type calibrated thermocouple with a diameter of 0.8 mm was placed nearly one mm from the middle of the hot wire to measure the temperature at the center of the sample. A T‐type thermocouple was attached to the brass cylinder containing the sample to measure the temperature at the outer surface. A multi‐channel data reader (CHY502A) was used to record the thermocouple data. The samples were placed inside the cylinder for testing, and both the cylinder and the samples were kept in an oven with basic temperature for two hours to reach thermal equilibrium. When both the thermocouples inside the samples and the outside of the cylinder containing the sample showed the same temperature, the direct current was established in the linear heat source. A digital multimeter was used to measure the current intensity of the circuit. Power consumption varied from 2.5 to 6 Wm^‐1^, which increased the temperature of samples between 5 to 14°C. Changes in the temperature of thermocouple were recorded in 3 s rounds by the data reader. The temperatures recorded by the thermocouple were then plotted against the normal logarithm of the time elapsed during the experiment. Slope (*S*) and the coefficient of determination (*R* ^2^) were determined sequentially for each experiment. The slope of the line drawn on the graph with the highest coefficient of determination value was used to calculate the thermal conductivity.

(23) $$k=\frac{I^{2}R}{4\pi S}$$

#### 2.2.4 Statistical analyses

In the present study, each experiment was performed in three replicates. The nonlinear regression method was used for fitting data and predicting variations in weight loss of Toast bread during baking. Goodness of fit was determined by coefficient of determination (*R* ^2^). Higher *R* ^2^ value indicates better fit.

## 3 RESULTS AND DISCUSSION

The results of chemical tests on wheat flour used to make toast bread are summarized in Table 2.

**TABLE 2:** Chemical properties of wheat flour used in toast bread

	Moisture (%)	Ash (%)	Protein (%)	Fat (%)	pH	Wet gluten (%)
Wheat flour	11.29	0.45	11.20	0.93	5.70	28.1

### 3.1 The changes crust temperature and weight loss of dough during the baking process

**FIGURE 1:**
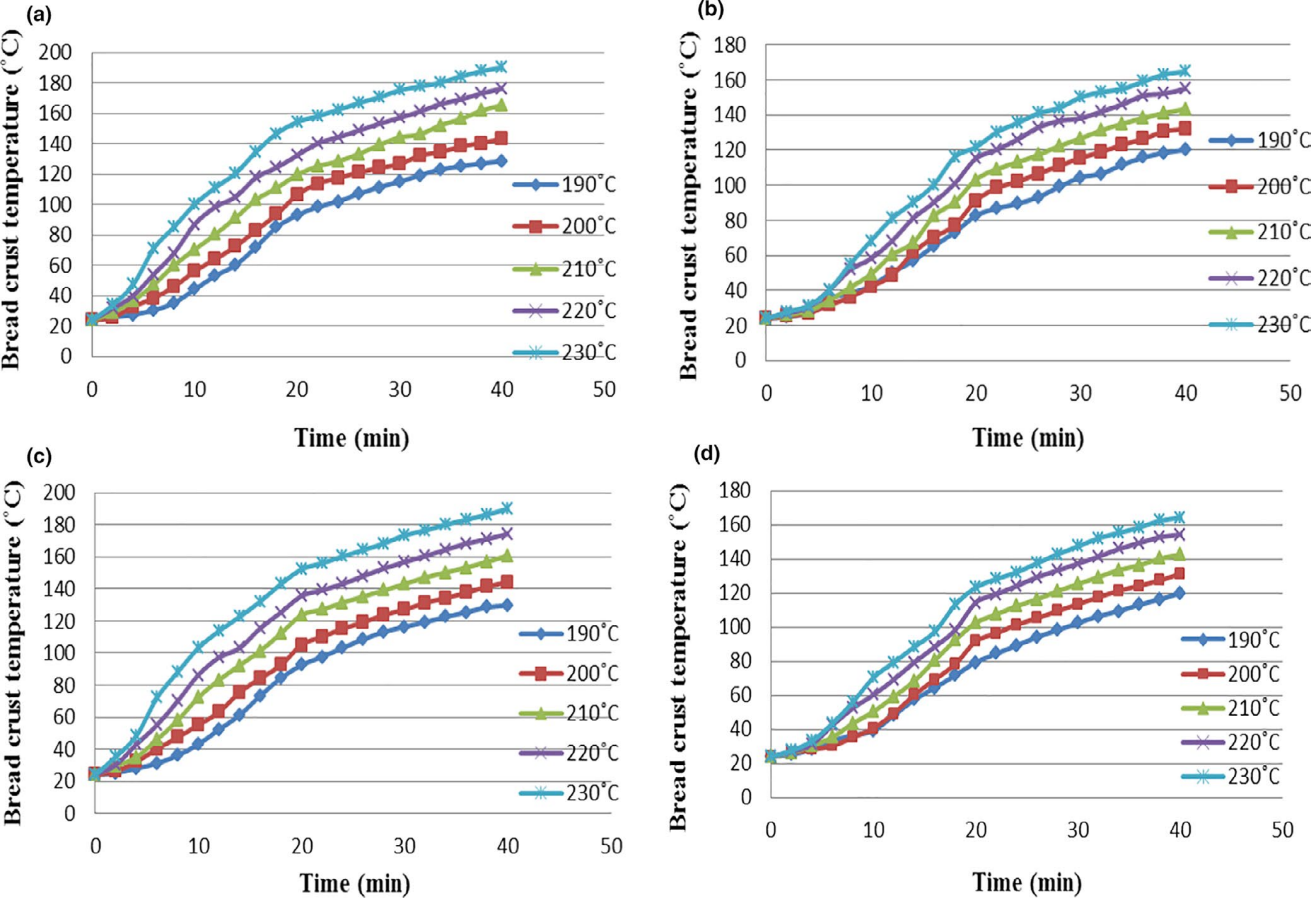
Predicted and experimental temperature of the Toast bread crust at different oven temperature. (a) predicted temperature of control; (b) predicted temperature of bread containing guar gum; (c) experimental temperature of control; (d) experimental temperature of bread containing guar gum

**FIGURE 2:**
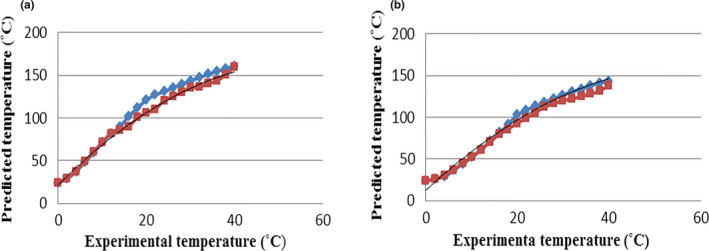
Comparison between experimental and predicted crust temperature of the toast breads. (a) control; (b) toast bread crust containing guar gum

The predicted changes and the experimental data are shown in Figure 1, related to the crust temperature of toast bread samples in the control and test (containing 1% of guar gum) groups, at different temperatures and baking durations. According to the results, in all bread samples, when the oven temperature increases, the crust temperature of the samples will rise and this increase is higher in samples of the control group than the test group. So that with increasing the oven temperature from 190°C to 230°C, the crust temperature of samples in the control group will rise from 128.5°C to 190.2°C, and of the toast bread samples in the test group from 120.18 to 164.8°C. With increasing baking duration, the crust temperature of bread samples will also rise. So that in the toast bread samples without guar gum, by increasing the baking duration from 0 to 40 min, the crust temperature will rise from 24 to 128.5°C, 24 to 143.17°C, 24 to 163.5°C, 24 to 176.21°C, and 24 to 190.2°C, respectively in ovens at temperatures of 190, 200, 210, 220, and 230°C. On the other hand, in toast bread samples containing guar gum, with increasing baking duration in the same temperature range of the oven, the corresponding increases of the temperature from 24 to 120.18°C, 24 to 132.15°C, 24 to 143.5°C, 24 to 155°C, and 24 to 164.8°C will be the result. Notably, during baking, the crust temperature of each toast bread samples containing guar gum, which has been cooked at temperatures of 190, 200, 210, 220, and 230°C, was reached respectively to 80, 81, 83, 85, and 88 percent of the oven temperature, with corresponding 81, 83.5, 85.5, 87.25, and 88.38 percent for samples of the control group. Generally, when the bread is placed in the oven, the crust temperature of the bread rises rapidly because of the rapid decline in the water content, while the changes are slower in the interior parts of the samples. But in the case of crumbs, the temperature reaches around 100°C and varies with a flat slope due to the presence of water until the end of the baking process (Bikard et al., 2008; Purlis, 2010, 2012). As the temperature rises, the partial vapor pressure in the pores increases and moisture is vaporized into the interior areas to maintain equilibrium. On the other hand, with increasing temperature, the partial vapor pressure in the pores rises, with higher values in the colder and inner parts of the bread and the moisture is vaporized into the inner areas to reach the equilibrium pressure. The outer layer of the bread is hotter and unsaturated making the surface moisture to diffuse outside and the surface to be dried. The moisture also diffuses from crumb to the crust in liquid form due to the concentration gradient between the interior and surface, but the content of water in the crumb is higher because of a higher diffusion rate of vapor than water. In this way, the diffused vapors are condensed in the cold interior areas (Khater & Bahnasawy, 2014; Zhang & Datta, 2006). As with results, bread samples containing guar gum has a lower crust temperature than bread samples of the control group. Importantly, the crust color of samples containing guar gum was lighter with better sensory quality after the baking process than the samples in the control group, due to the enhanced browning reaction caused by the addition of gum. Because the addition of hydrocolloids enhances the intensity of the Maillard reaction by affecting water diffusion and increasing the reaction between the nitrogen complex of glycosylamine and the reducing carbohydrates (Lazaridou et al., 2007). Conversely, Purlis (2011) reported that during the baking inside the oven, 69%, 28%, and 3% of the heat absorbed by the dough is contributed in respectively by radiation, diffusion, and thermal conductivity. Therefore, considering the lighter surface of guar gum‐containing samples than the control ones, the lower temperature of crust in this group is justifiable. The predicted and experimental data are shown in figure 2 for comparison. Then, Equations (24) and (25) were calculated using nonlinear regression analysis to express the relationship between the predicted and experimental crust temperatures respectively for control and test samples Figures 2.

(24) $$T_{\mathrm{prc}}=‐0.0462{\left(T_{\mathrm{Ex}}\right)}^{2}+5.1542T_{\mathrm{Ex}}+21.62\;R^{2}=0.98$$

(25) $$T_{\mathrm{prt}}=‐0.0434{\left(T_{\mathrm{Ex}}\right)}^{2}+5.074T_{\mathrm{Ex}}+12.02\;R^{2}=0.98$$

**FIGURE 3:**
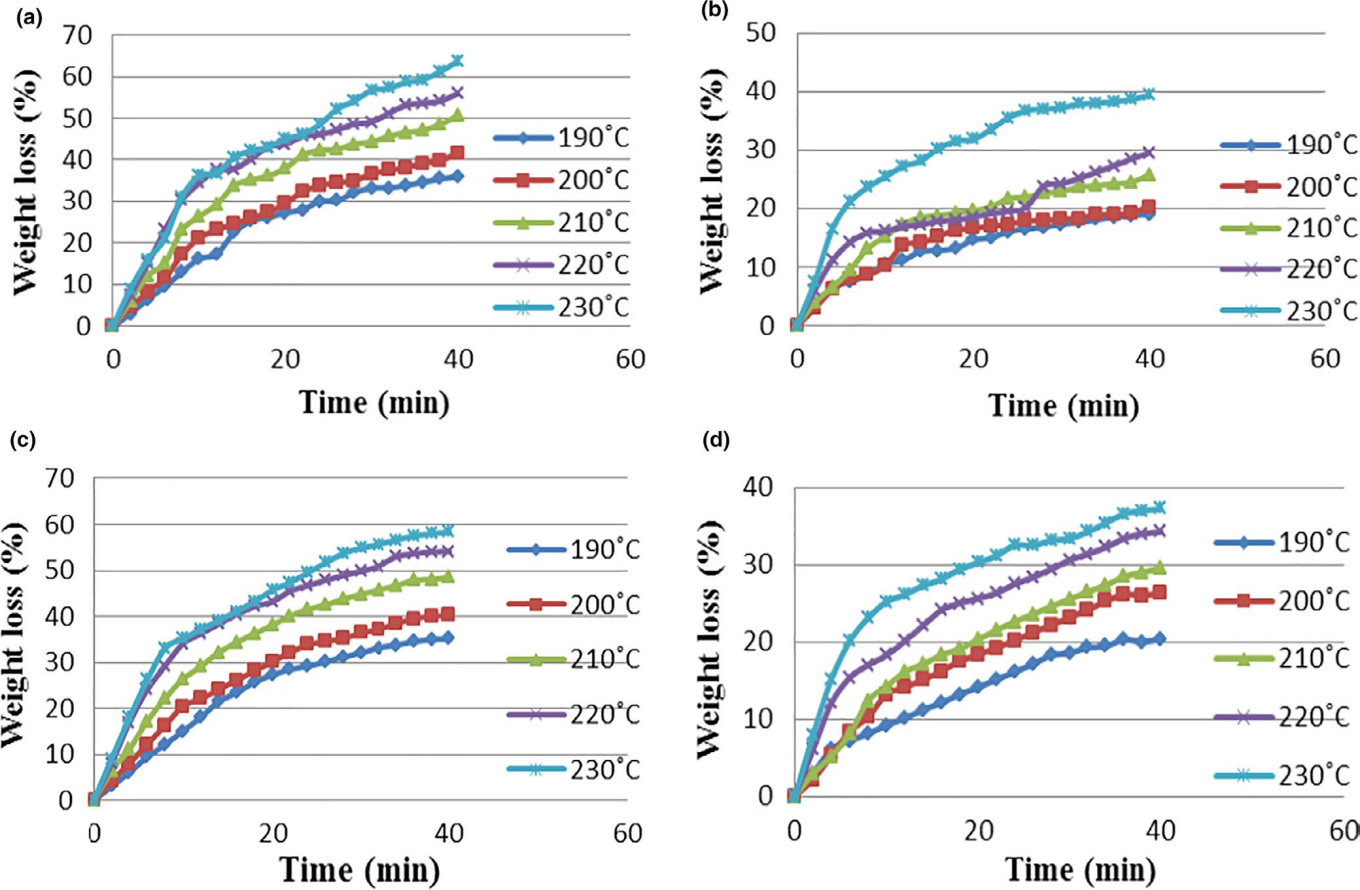
Predicted and experimental temperature of weight loss of bread at different oven temperature. (a) predicted weight loss of control; (b) predicted weight loss of bread containing guar gum; (c) experimental weight loss of control; (d) experimental weight loss of bread containing guar gum

**FIGURE 4:**
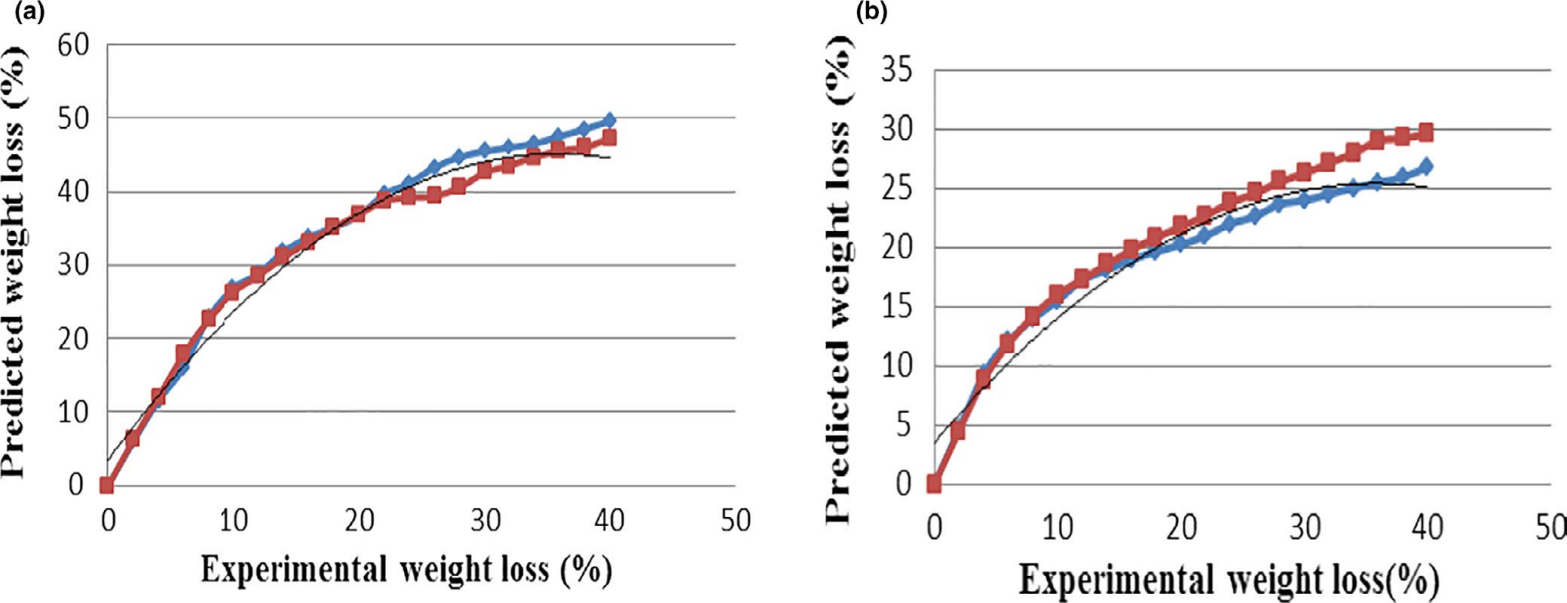
Comparison between experimental and predicted weight loss of the Toast breads. (a) Control; (b) toast bread weight loss containing guar gum

The predicted changes and the experimental data are shown in Figures 3 and 4, related to the percentage of weight loss of toast bread samples in the control and test (containing 1% of guar gum) groups, at different temperatures and baking durations. According to the results, when the oven temperature increases, the percentage of weight loss in samples will raise and this increase is higher in samples of the control group than the test group. So that with increasing the oven temperature from 190°C to 230°C, the percentage of weight loss of samples in the control group will raise from 35.11% to 47.23%, and of the toast bread samples in the test group from 20.37% to 29.57%. With increasing baking duration, the percentage of weight loss of bread samples will also raise. So that in the toast bread samples without guar gum, by increasing the baking duration from 0 to 40 m, the percentage of weight loss will rise from 0% to 35.11%, 0 to 40.25%, 0 to 48.47%, 0 to 54.05%, and 0 to 58.31%, respectively in ovens at temperatures of 190, 200, 210, 220, and 230°C. On the other hand, with increasing baking duration in the same temperature range of the oven, the corresponding increases of the weight loss in toast bread samples containing guar gum of 0 to 20.37%, 0 to 26.41%, 0 to 29.57%, 0 to 34.27%, and 0 to 37.25% will be the result. According to the results, at the start of the baking process, the moisture content was decreased with a steep (and ultimately with a gentle) slope; because at the start of baking, the moisture on the bread surface is evaporated more rapidly (especially at high temperatures) than the other areas. Therefore, due to the hardening of the bread crust during the initial baking duration, the evaporation rate gradually diminished over time (Khater & Bahnasawy, 2014; Zhang & Datta, 2006). Conversely, as the baking temperature increases, the slope of the partial moisture pressure between the bread surface and the hot air inside the oven rises; under this condition, the coefficient of effective moisture diffusion inside the bread is increased, resulting in a decline in the weight loss of the bread samples. Importantly, the percentage of weight loss in samples containing guar gum was lower than that of the control samples, because the addition of guar gum will increase the moisture content of the bread samples due to the presence of hydroxyl groups in these compounds that form hydrogen bonds with water, leading to the stability of the dough's gluten network, better maintenance of dough water, reduced rate of staling and stiffness of the product (Lazaridou et al., 2007; Movahhed & Mirzaei, 2013). Then, Equations (26) and (27) were calculated using nonlinear regression analysis to express the relationship between predicted and experimental percentages of weight loss respectively for samples of the control and test groups.

(26) $$WL_{\mathrm{prc}}=‐0.0325{\left(Wl_{\mathrm{Ex}}\right)}^{2}+2.326Wl_{\mathrm{Ex}}+3.474\;R^{2}=0.99$$

(27) $$WL_{\mathrm{prt}}=‐0.017{\left(Wl_{\mathrm{Ex}}\right)}^{2}+1.218Wl_{\mathrm{Ex}}+3.507\;R^{2}=0.99$$

## 4 CONCLUSION

The present research investigated the impact of guar gum, and different oven temperatures and baking times on weight loss and the temperature of bread crust of the toast bread samples. According to the results, in all samples, the temperature of the crust of bread was increased by increasing the oven temperature and the increase in temperature of control samples (48.01%) was more than samples with guar gum (37.12%). Besides, by increasing the baking time, the temperature of the crust was increased and control samples experienced more temperature increase than samples with guar gum. As per the results, in control samples and samples with guar gum by increasing the oven temperature from 190 to 230°C, the crust temperature was increased from 81% to 88.38% and from 80% to 88%, respectively. On the other hand, by increasing the oven temperature from 190 to 230°C, the percentage of weight loss in samples was increased from 35.11% to 47.23% in control samples and from 20.37% to 29.57% increased in samples with guar gum. Also, by increasing the baking time from zero to 40 min and in all oven temperatures from 190 to 230°C, control samples lost a considerable amount of weight. It is suggested that future researches can study the improving agent and at different temperatures and times. Furthermore, for more precise modeling, measure the changes of volume, porosity, and density of toast bread. The predicted bread crust temperature and weight loss of bread (control and containing guar gum) were in a reasonable agreement with the experimental temperature with a coefficient of determination of 0.98 and 0.99, respectively.

## Nomenclature

*a_w_*: Water activity, dimensionless
*ρ*: Dough density, kg m^‐3^
*S_c_*: Schmidt number, dimensionless
*T_cw_*: Cold water temperature, K
*S_sat_*: Water vapor pressure at saturation, Pa
RH: Relative humidity, %
*P_r_*: Prandtl number, dimensionless
$M_{\mathrm{hw}}$: Mass of hot water, kg
T: Temperature of bread at as a function of time, K
t: Time, s
υ: Kinematic viscosity, m^2^ s^‐1^
$T_{\mathrm{hw}}$: Hot water temperature, K
$P_{\infty }$: Water vapor pressure of the air at the oven ambient, Pa
r: Bread radius, m
α: Thermal diffusivity, m^2^ s^‐1^
$H_{c}$: Heat capacity of container, J K^‐1^
$P_{s}$: Water vapor pressure of the air at the bread surface, Pa
$T_{s}$: Surface temperature, K
$k_{g}^{*}$: Mass transfer coefficient, kg pa^‐1^m^‐2^ s^‐1^
$T_{c}$: Temperature of container, K
$C_{P}$: Specific heat of bread, J kg^‐1^ K^‐1^
$T_{\infty }$: Ambient temperature, K
ε: Emissivity, dimensionless
$T_{m}$: Dough temperature, K
σ: Stefan–Boltzmann constant, dimensionless
$k_{g}$: Corrected mass transfer coefficient, kg Pa^‐1^ m^‐2^ s^‐1^
$H_{f}$: Heat capacity of flax, J K^‐1^
$M_{m}$: Dough mass, kg
$\lambda_{v}$: Latent heat of evaporation, J kg^‐1^
D: Water diffusion coefficient, m^2^ s^‐1^
$M_{\mathrm{cw}}$: Mass of cold water, kg
R: Resistance, Ω
k: Bread thermal conductivity, W m^‐1^ K^‐1^
W: Water content of bread, kg kg^‐1^
$C_{w}$: Heat capacity of water, J K^‐1^
S: Slope of the linear portion of the plot of the temperature vs. ln(time)
h: Heat transfer coefficient, W m^‐2^ K^‐1^
$\rho_{s}$: Density of solid, kg m^‐3^
$T_{e}$: Equilibrium temperature of cold water, K
I: Current through wire, A
${\mathrm{WL}}_{\mathrm{prt}}$: Predicted weight loss of sample containing guar, %
${\mathrm{Wl}}_{\mathrm{Ex}}$: Measured weight loss, %
$T_{\mathrm{prc}}$: Predicted temperature of control, °C
$T_{\mathrm{prt}}$: Predicted temperature of sample containing guar, °C
$T_{\mathrm{Ex}}$: Measured bread temperature, °C
${\mathrm{WL}}_{\mathrm{prc}}$: Predicted weight loss of control, %

## CONFLICT OF INTEREST

The authors declare that they do not have any conflict of interest.

## ETHICAL APPROVAL

This study does not involve any human or animal testing.

## Data Availability

The data that support the findings of this study are openly available at https://doi.org/10.1002/fsn3.1993.
